# The dynamics of frailty development and progression in older adults in primary care in England (2006–2017): a retrospective cohort profile

**DOI:** 10.1186/s12877-021-02684-y

**Published:** 2022-01-06

**Authors:** Carole Fogg, Simon D. S. Fraser, Paul Roderick, Simon de Lusignan, Andrew Clegg, Sally Brailsford, Abigail Barkham, Harnish P. Patel, Vivienne Windle, Scott Harris, Shihua Zhu, Tracey England, Dave Evenden, Francesca Lambert, Bronagh Walsh, The Frailty Dynamics study team

**Affiliations:** 1 School of Heath Sciences, Faculty of Environmental and Life Sciences, University of Southampton, University Road, Southampton, SO17 1BJ UK; 2 School of Primary Care, Population Sciences, and Medical Education, Faculty of Medicine, Southampton General Hospital, Tremona Road, Southampton, SO16 6YD UK; 3 Nuffield Department of Primary Care Health Sciences, University of Oxford, Eagle House, Walton Well Road, Oxford, OX2 6ED UK; 4 Royal College of General Practitioners, Research and Surveillance Centre, 30, Euston Square, London, NW1 2FB UK; 5 Academic Unit for Ageing & Stroke Research, University of Leeds, Bradford Teaching Hospitals NHS Foundation Trust, Duckworth Lane, Bradford, BD9 6RJ UK; 6 Southampton Business School, University of Southampton, University Road, Southampton, SO17 1BJ UK; 7 Southern Health NHS Foundation Trust, Unit 1 Wessex Way, Colden Common, Winchester, SO21 1WP UK; 8 Medicine for Older People, University Hospitals Southampton NHS Foundation Trust, Southampton General Hospital, Tremona Road, Southampton, SO16 6YD UK; 9 NIHR Southampton Biomedical Research Centre, Southampton Centre for Biomedical Research, Southampton General Hospital, Tremona Road, Southampton, SO16 6YD UK

**Keywords:** Frailty, Cohort study, Adults, Trajectories, Computer simulation modelling, Primary care, Service use, Electronic health records

## Abstract

**Background:**

Frailty is a common condition in older adults and has a major impact on patient outcomes and service use. Information on the prevalence in middle-aged adults and the patterns of progression of frailty at an individual and population level is scarce. To address this, a cohort was defined from a large primary care database in England to describe the epidemiology of frailty and understand the dynamics of frailty within individuals and across the population. This article describes the structure of the dataset, cohort characteristics and planned analyses.

**Methods:**

Retrospective cohort study using electronic health records. Participants were aged ≥50 years registered in practices contributing to the Oxford Royal College of General Practitioners Research and Surveillance Centre between 2006 to 2017. Data include GP practice details, patient sociodemographic and clinical characteristics, twice-yearly electronic Frailty Index (eFI), deaths, medication use and primary and secondary care health service use. Participants in each cohort year by age group, GP and patient characteristics at cohort entry are described.

**Results:**

The cohort includes 2,177,656 patients, contributing 15,552,946 person-years, registered at 419 primary care practices in England. The mean age was 61 years, 52.1% of the cohort was female, and 77.6% lived in urban environments. Frailty increased with age, affecting 10% of adults aged 50–64 and 43.7% of adults aged ≥65. The prevalence of long-term conditions and specific frailty deficits increased with age, as did the eFI and the severity of frailty categories.

**Conclusion:**

A comprehensive understanding of frailty dynamics will inform predictions of current and future care needs to facilitate timely planning of appropriate interventions, service configurations and workforce requirements. Analysis of this large, nationally representative cohort including participants aged ≥50 will capture earlier transitions to frailty and enable a detailed understanding of progression and impact. These results will inform novel simulation models which predict future health and service needs of older people living with frailty.

**Study registration:**

Registered on www.clinicaltrials.gov October 25th 2019, NCT04139278.

**Supplementary Information:**

The online version contains supplementary material available at 10.1186/s12877-021-02684-y.

## Introduction

Frailty is a state of vulnerability to minor stressors such as infections or falls, which leads to a disproportionate decline in overall health status and increased risk of disability and death [1, 2]. Frailty can be identified and classified by severity using either a phenotype model following a physical assessment [3], or by using a frailty score or ‘index’ based on an accumulation of conditions or deficits, which can be derived from routinely collected data or other longitudinal surveys of health status [4]. Pooled analysis of European data estimates the prevalence of frailty in older adults at 18% in all settings, and 12% in community-based studies [5]. Frailty is more common as age increases but is not synonymous with ageing; evidence suggests increased variability in frailty with ageing, but age only partly explains frailty trajectories [6, 7]. Factors including sociodemographics, specific long-term conditions, physical activity and education have been associated with frailty progression [8]. It has been estimated that a doubling in deficits (and therefore the frailty score) occurs over 12.6 years, although the small cohort size suggests further confirmation is needed [9]. There is still uncertainty as to the relationship between the rate of change of deficit accumulation and death [10, 11]. Current evidence is predominantly cross-sectional and there is contradictory evidence from studies with different methods of assessing frailty and varying analytical approaches, necessitating standardisation of study methods and greater use of longitudinal cohorts [8]. In addition, little is known about the onset of frailty and its development in middle-aged and younger old adults and the impact of frailty transitions in these age groups within typical primary care populations, making population health and social care planning across this part of the life-course difficult, given that frailty is already prevalent in people by the age of 65 [9].

Although frailty research has expanded over the last decade, use of different measures and concepts of frailty, small cohort sizes that are not representative of the wider older population, and variable lengths of follow-up have led to uncertain conclusions on the occurrence and progression of frailty within ageing populations [12], particularly in primary care. There is therefore a need for larger, longer cohort representative studies which include standardised methods of establishing frailty, with key covariates related to frailty risk and risk of other adverse outcomes and clear descriptions of populations and methods. An electronic Frailty Index (eFI) has been developed and validated using routine primary care data and has demonstrated robust predictive validity for outcomes of mortality, hospitalisation and nursing home admission, outcomes associated with the vulnerabilities attributed to frailty, and has the ability to detect longitudinal changes at an individual level [13, 14]. The use of the eFI as a risk stratification tool is expanding and has become standard throughout primary care in England, making the eFI an appropriate measure for epidemiological studies of frailty in primary care at population level. As the eFI score can be calculated from routine primary care records, it is possible to apply the tool to the study of large datasets to generate widely understood and transferable findings [15, 16].

The cohort described in this paper is part of a larger project which aims to describe the epidemiology and dynamics of frailty in the adult primary care population (aged ≥50 years) and its impact on patients and health and social care services and costs [17]. These analyses will inform the development of a System Dynamics simulation model to predict future trends in frailty and related service demand for the purpose of developing guidelines and tools to facilitate commissioning and service development [18].

This paper gives an overview of the cohort dataset and key variables, and presents baseline descriptive data both for the general practices from which patient records originated and for the participants.

## Methods

### Study design

Retrospective cohort using routinely collected electronic health records.

### Study setting

The UK has a registration-based primary healthcare system where patients are registered with a single general practice. Patients are allocated a personal lifetime unique identifier, (a National Health Service (NHS) number), which reduces the risk of duplicate records and facilitates the linkage of primary care to other healthcare datasets. The Oxford-Royal College of General Practitioners (RCGP) Research and Surveillance Centre (RSC) is a network of around 5% of primary care practices in England which contribute electronic health record data voluntarily. Practices registered with the RCGP RSC have been shown to be nationally representative in terms of the population served and health outcomes [19, 20].

### Eligibility criteria

The inclusion criteria were patients (i) aged ≥50 years, (ii) registered at a general practice contributing to the Oxford-RCGP RSC network database and (iii) registered at any time between 2006 to 2017. Potential duplicate patients were excluded, i.e. more than one sex present for a patient record, duplicated calendar years of data and differing birthdates in the patient record, and patients with missing or impossible birthdates. Yearly records were excluded as follows: (i) where a patient changed practice within a calendar year and had duplicate yearly records, the yearly record with the longest period was kept if continuous years of data were available; (ii) person-years of data following a gap of 1 year or more in the observation record, even if the patient re-registered with an RCGP RSC practice.

Data from all participants constitutes the open cohort – i.e. a cohort which included entry of new registrants such as patients turning 50 and those entering RCGP RSC practices from other areas. A closed cohort was also defined, including only eligible participants present in the cohort index year of 2006. These two cohorts will be used for different analyses during the project and allow for exploration of cohort ageing and the impact of frailty on the overall population on service use.

### Data sources

Electronic health records (EHR) were extracted from the RCGP RSC dataset. Publicly available datasets were linked to the primary care data, including the Income Deprivation Affecting Older People Index (IDAOPI) 2015 [21], geographical information from the geography portal of the Office for National Statistics (ONS) [22] and workforce data for GP practices [23].

### Measures

#### Electronic frailty index

The electronic frailty index (eFI) was used to identify and grade the severity of frailty [14]. The eFI includes 36 deficits across disease states, symptoms/signs, abnormal laboratory values and indicators of disability which were identified according to standard methods for creating a frailty index [24]. An eFI score is derived as the number of deficits present as an equally weighted proportion of the total possible [14]. Using the same Read codes (Clinical Terms Version 3 - CTV3) as in the original derivation of the score, variables for each deficit were created and flagged as ‘present’ if the Read codes were present in the patient EHR at any point in their prior medical history on the 1st January and 1st July for each calendar year for each participant. As this method retrieves codes from the patient’s complete medical record, there is no missing data for any of the deficits. An eFI score (total deficits at each 6-monthly cut-point/36) was then generated, and categorised into frailty states indicating increasing severity and risk of poor outcomes as: fit (0–0.12), mild (0.13–0.24), moderate (0.25–0.36) or severe (> 0.36) [14]. As the eFI is designed to be a cumulative index, reversals (for example data artefacts due to change in GP practice during follow-up) were imputed to the previous frailty state, apart from the polypharmacy deficit, which is calculated from prescription information from the previous 12 months.

#### Sociodemographic variables

Sociodemographic variables include age, sex, Indices of Multiple Deprivation (IMD) quintiles and IDAOPI quintiles. The IMD is a small-area measure of socioeconomic status based on postcode, ranked nationally, which includes seven domains: income, employment, education/skills/training, health and disability, crime, barriers to housing and services, and living environment [25]. The IDAOPI is a subset of the Income Deprivation Domain, and focusses specifically on the percentage of the population aged 60 and over who receive income support, income based job seekers allowance, pension credit or child tax credit claimants aged 60 and over and their partners aged ≥60. The 2015 deprivation indices were related to the last known patient address in the dataset or, where missing, were imputed using the IMD or IDAOPI indices related to the GP practice address (3.6% of patients). Ethnicity data was maximised using a customised ontology and coded into categories (Asian, Black, White, Mixed/other) [26]. The most recent ethnicity reported in the patient record was used as the baseline ethnicity value to reduce missing values in the year of entry to the cohort. Age was categorised into four groups, reflecting groupings reported in literature relating to older adults’ healthcare, and cut-offs for services reported by the study Stakeholder Engagement Group (SEG): 50–64, 65–74, 75–84 and ≥ 85. Receipt of residential care during the cohort period for each patient was coded ‘yes’ or ‘no’ by using a combination of Read codes [13] and use of a household key (11 or more patients at the same address with a median age of 50 or above) for the patient’s last known address at the date of data extraction (May 2019).

#### Clinical variables

Individual eFI deficits, associated specific common long-term condition codes (e.g. COPD, asthma, rheumatoid arthritis) not included in the eFI and those present in the Quality Outcomes Framework (QOF) (e.g. dementia, depression, cancer, obesity) with dates of onset, were generated on a yearly basis for each patient ascertained from the whole patient medical history. Other annual data included smoking status, influenza and pneumococcal vaccinations. All body mass index (BMI) measurements present in the patient record were provided. Due to the differences in availability of values, a baseline BMI value was defined as the first recording in a patient’s cohort entry year, or, where missing, the first value in the nearest previous year to cohort entry (up to a maximum of 2 years) or the nearest year afterwards (up to 2 years).

#### General practice variables

General practice information included the geographical region, urban/rural indicators based on the 2011 rural/urban classification (RUC11) [27], IMD and IDAOPI for the practice postcode, number of patients registered in the practice, and total practice consultations per year. The total general practitioner (GP), nurse and overall practice staff full-time equivalent (FTE) for each general practice in 2013 (the first year this information is available to be linked on practice code) was included [28]. Each calendar year of participant data was linked to a general practice identifier and dates of the participant registering and leaving the RCGP RSC practice were provided.

#### Death

The month and year of death were provided. Primary care death data in the RCGP dataset has been shown to be accurate for this calendar period [29, 30].

#### Service use

Primary care service use outcomes include number of days in a calendar year with a consultation by consultation type (administrative, face-to-face, telephone, electronic consultation, or home visit), total number of GP prescriptions and number of unique prescriptions by British National Formulary (BNF) chapter [31].

### Statistical analysis

A description of the primary care data from the open cohort is presented in this paper. The characteristics of RCGP RSC practices with participants in the cohort are described for the calendar year 2006 (first year of cohort). Age category distributions for both the open and closed cohorts were analysed and presented graphically for the calendar years 2006–2017. The reasons for exit from the cohort are summarised. Patient sociodemographic and clinical characteristics at the year of cohort entry (i.e. for the open cohort) are described according to the four age groups, and missing data quantified.

## Results

**Fig. 1:**
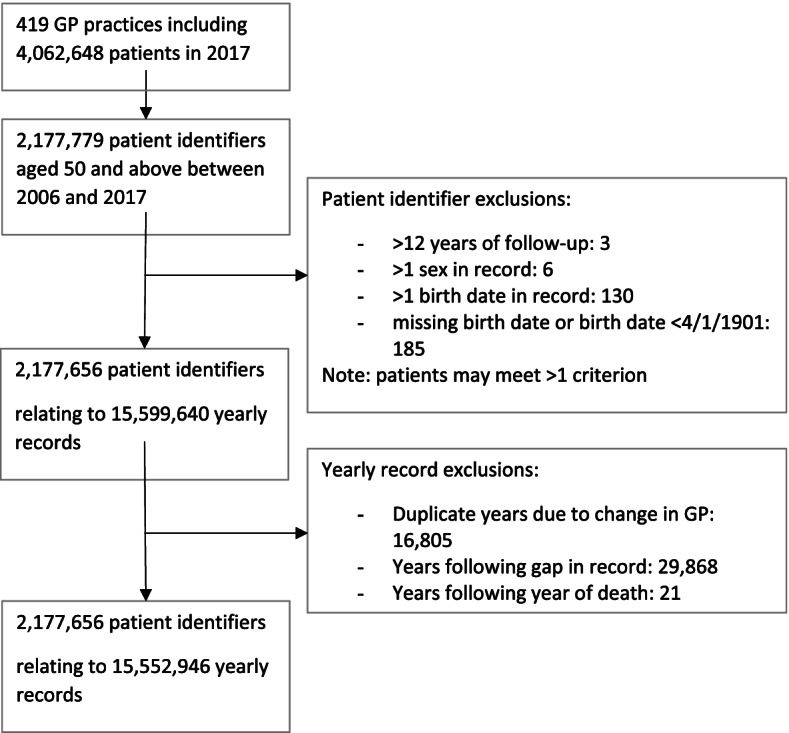
Cohort definition

The open cohort comprised 2,177,656 patients, contributing 15,552,946 person-years of data (Fig. 1).

### Practice characteristics

Four hundred and nineteen primary care practices distributed across England contributed to the cohort between 2006 and 2017 inclusive. Practices varied widely in their patient numbers and consequently their totals of yearly consultations (Table 1). Practices reflected population distributions throughout England, with 78% in urban areas and an even spread across IMD quintiles.

**Table 1:** Primary care practice characteristics in 2006 (*n* = 419)

Practice characteristic	n (%)
Geographic region (n, %)	
London	64 (15.3%)
Midlands and East	90 (21.5%)
North	128 (30.6%)
South	137 (32.7%)
Rural/urban Classification	
Urban: Major conurbation	150 (35.8%)
Urban: Minor conurbation	15 (3.6%)
Urban: City and Town	163 (38.9%)
Rural: Town and fringe	69 (16.5%)
Rural: Village and dispersed	22 (5.3%)
Practice size (patients)	
median	6858
Upper: lower quartile	4110: 9819
Practice size (staff - FTEs)^a^	
GPs (mean, SD)	6 (3)
Nurses^b^ (mean, SD)	2 (2)
Total staff^b^ (mean, SD)	14 (8)
Consultations (median, upper: lower quartile)	
Face to face	42,661 [22,426: 72,362]
Clinical administration	20,474 [5811: 73,050]
e-consultations (data from 2017)	4 [0: 43]
Telephone	1713 [261: 4383]
Home visits	669 [50: 1992]
Practice Index of Multiple Deprivation quintile	
Most deprived	93 (22.2%)
2nd quintile	84 (20.1%)
3rd quintile	83 (19.8%)
4th quintile	85 (20.3%)
Least deprived	74 (17.7%)

^a^Data not available for 2 practices

^b^Not specified in 30 practices

### Patient baseline characteristics

The sociodemographic and clinical baseline characteristics of participants in their year of entry to the cohort are presented in Tables 2 and 3. The mean age of participants was 61 years (SD 12). Demographic trends with increasing age were observed, including a higher proportion of female sex, lower ethnic diversity and rural residence in the older age groups. Ethnicity data was more likely to be missing with increasing age, decreasing deprivation, male sex, urban location and for people in residential care. Patterns of indices of deprivation appeared similar across age groups, with half the cohort located in the two least deprived quintiles. Long-term conditions were more prevalent in older age groups at baseline, with the exception of depression and obesity which were more common in younger age groups. The eFI score increased with age, as did the proportion of participants in the mild, moderate and severe frailty categories. The proportion of people with frailty at cohort entry increased from 10% in the 50–64 age group to 69% in people aged ≥85.

**Table 2:** Participant sociodemographic baseline characteristics

	Age Group				Total (%)
	50–64	65–74	75–84	≥85	
**Age group** ^a^	1,413,576 (64.9%)	385,474 (17.7%)	259,125 (11.9%)	119,481 (5.5%)	2,177,656
**Female**	698,158 (49.4%)	199,914 (51.9%)	151,462 (58.5%)	84,437 (70.7%)	1,133,971 (52.1%)
**Ethnicity** ^b^					
Asian	52,703 (5.1%)	11,419 (4.1%)	4521 (2.7%)	916 (1.4%)	69,559 (4.5%)
Black	29,387 (2.8%)	5577 (2.0%)	2350 (1.4%)	440 (0.7%)	37,754 (2.4%)
Mixed/Other	15,461 (1.5%)	2480 (0.9%)	1110 (0.6%)	277 (0.4%)	19,328 (1.3%)
White	937,135 (90.6%)	260,473 (93.0%)	160,063 (95.3%)	63,054 (97.5%)	1,420,725 (91.8%)
*Missing*^*c*^	*378,890 (26.8%)*	*105,525 (27.4%)*	*91,081 (35.2%)*	*54,794 (45.9%)*	*630,290 (28.9%)*
**Urban**	1,102,809 (78.0%)	294,247 (76.3%)	200,358 (77.3%)	91,492 (76.6%)	1,688,906 (77.6%)
**Residential care**	1019 (0.1%)	1708 (0.4%)	5371 (2.1%)	9121 (7.6%)	17,219 (0.8%)
**IMD quintile**					
1 (Most deprived)	193,552 (13.7%)	49,320 (12.8%)	34,151 (13.2%)	14,894 (12.5%)	291,917 (13.4%)
2	220,674 (15.6%)	60,287 (15.6%)	41,887 (16.2%)	19,592 (16.4%)	342,440 (15.7%)
3	280,969 (19.9%)	79,288 (20.6%)	54,244 (20.9%)	25,806 (21.6%)	440,307 (20.2%)
4	340,796 (24.1%)	93,998 (24.4%)	62,573 (24.2%)	28.815 (24.1%)	526,182 (24.2%)
5 (Least deprived)	377,585 (26.7%)	102,581 (26.6%)	66,270 (25.6%)	30,374 (25.4%)	576,810 (26.5%)
**IDAOPI quintile**					
1 (Most deprived)	199,722 (14.1%)	50,167 (13.0%)	34,440 (13.3%)	15,493 (13.0%)	299,822 (13.8%)
2	217,183 (15.4%)	58,934 (15.3%)	42,894 (16.6%)	19,930 (16.7%)	338,941 (15.6%)
3	269,450 (19.1%)	76,828 (19.9%)	55,166 (21.3%)	27,233 (22.8%)	428,677 (19.7%)
4	336,857 (23.8%)	93,684 (24.3%)	62,160 (24.0%)	29,063 (24.3%)	521,764 (24.0%)
5 (Least deprived)	390,364 (27.6%)	105,861 (27.5%)	64,465 (24.9%)	27,762 (23.2%)	588,452 (27.0%)

^a^% as proportion of total cohort

^b^% as proportion of known values

^c^missing values as % of cohort

**Table 3:** Participant baseline clinical characteristics

	Age Group				Total (%)
	50–64	65–74	75–84	≥85	
**eFI score**					
median	0.028	0.083	0.139	0.167	0.056
Upper: lower quartile	[0: 0.083]	[0.028: 0.139]	[0.083: 0.194]	[0.111: 0.250]	[0.028: 0.111]
**Frailty category**					
Fit	1,273,304 (90.1%)	272,694 (70.7%)	120,357 (46.5%)	37,243 (31.2%)	1,703,598 (78.2%)
Mild	127,029 (9.0%)	94,558 (24.5%)	99,154 (38.3%)	49,192 (41.2%)	369,933 (17.0%)
Moderate	12,055 (0.9%)	16,167 (4.2%)	32,732 (12.6%)	25,360 (21.2%)	86,214 (3.4%)
Severe	1188 (0.1%)	2055 (0.5%)	6882 (2.7%)	7686 (6.4%)	17,811 (0.8%)
**Long-term conditions**					
Atrial fibrillation	11,359 (0.8%)	15,381 (4.0%)	23,978 (9.3%)	17,553 (14.7%)	68,271 (3.1%)
Coronary Artery Disease	16,176 (1.1%)	16,017 (4.2%)	12,015 (4.6%)	2419 (2.0%)	46,627 (2.1%)
Dementia	7705 (0.6%)	7812 (2.0%)	18,748 (7.2%)	19,328 (16.2%)	53,593 (2.5%)
Depression	271,343 (19.2%)	55,438 (14.4%)	37,418 (14.4%)	18,220 (15.3%)	382,419 (17.6%)
Haemorrhagic Stroke	3938 (0.3%)	1959 (0.5%)	1733 (0.7%)	867 (0.7%)	8497 (0.4%)
Heart Failure	6219 (0.4%)	8736 (2.3%)	14,976 (5.8%)	12,583 (10.5%)	42,514 (2.0%)
Hypertension	265,702 (18.8%)	161,622 (41.9%)	136,905 (52.8%)	60,133 (50.3%)	624,362 (28.7%)
Ischaemic Stroke	9833 (0.7%)	11,097 (2.9%)	15,836 (6.1%)	10,617 (8.9%)	47,383 (2.2%)
Malignancy	48,115 (3.4%)	32,230 (8.4%)	29,796 (11.5%)	14,646 (12.3%)	124,787 (5.7%)
Peripheral Arterial Disease	8144 (0.6%)	9541 (2.5%)	11,073 (4.3%)	4992 (4.2%)	33,750 (1.6%)
Rheumatoid Arthritis	11,149 (0.8%)	6244 (1.6%)	5236 (2.0%)	2169 (1.8%)	24,798 (1.1%)
Transient Ischaemic Attack	8065 (0.6%)	10,774 (2.8%)	15,795 (6.1%)	10,916 (9.1%)	45,550 (2.1%)
Diabetes	89,567 (6.3%)	49,954 (13.0%)	37,755 (14.6%)	13,514 (11.3%)	190,790 (8.8%)
Chronic Obstructive Pulmonary Disease	28,352 (2.0%)	22,538 (5.9%)	20,399 (7.9%)	7395 (6.2%)	78,684 (3.6%)
Chronic Kidney Disease	83,821 (5.9%)	42,059 (10.9%)	36,783 (14.2%)	19,404 (16.2%)	182,067 (8.4%)
Asthma	95,438 (6.8%)	31,682 (8.2%)	20,747 (8.0%)	6365 (5.3%)	154,232 (7.1%)
Osteoporosis	26,939 (1.9%)	21,884 (5.7%)	24,155 (9.3%)	14,096 (11.8%)	87,074 (4.0%)
Morbid obesity risk group	46,465 (3.3%)	9516 (2.5%)	3799 (1.5%)	697 (0.6%)	60,477 (2.8%)
**BMI category** ^a^					
Underweight	10,660 (1.2%)	4749 (1.6%)	6520 (3.5%)	5547 (8.8%)	27,476 (1.9%)
Normal	270,394 (29.3%)	88,178 (29.9%)	70,979 (37.9%)	31,659 (50.4%)	461,210 (31.4%)
Overweight	350,099 (38.0%)	119,969 (40.7%)	72,079 (38.4%)	18,858 (30.3%)	561,005 (38.2%)
Obese	290,704 (31.5%)	82,017 (27.8%)	37,970 (20.3%)	6743 (10.7%)	417,434 (28.5%)
*Missing* ^b^	*491,719 (34.8%)*	*90,561 (23.5%)*	*71,577 (27.6%)*	*56,674 (47.4%)*	*710,531 (32.6%)*
**Vaccinations**					
Flu vaccination	248,157 (17.6%)	269,364 (69.9%)	187,976 (72.5%)	71,906 (60.2%)	777,403 (35.7%)
Pneumococcal vaccination	119,926 (8.5%)	231,908 (60.2%)	184,638 (71.3%)	77,218 (64.6%)	613,690 (28.2%)
**Smoking status** ^a^					
Non-smoker	539,051 (40.7%)	138,073 (37.9%)	94,660 (39.7%)	51,381 (52.4%)	823,165 (40.6%)
Ex-smoker	437,970 (33.0%)	157,393 (43.2%)	109,868 (46.1%)	37,801 (38.5%)	743,032 (36.7%)
Active smoker	348,396 (26.3%)	68,858 (18.9%)	33,807 (14.2%)	8904 (9.1%)	459,965 (22.7%)
*Missing* ^b^	*88,159 (6.2%)*	*21,150 (5.5%)*	*20,790 (8.0%)*	*21,395 (17.9%)*	*151,494 (7.0%)*
**Prescriptions**					
median	4	18	32	39	8
Upper: lower quartile	[0: 15]	[4: 42]	[12: 60]	[16: 71]	[1: 29]

^a^% as proportion of known values

^b^missing values as % of cohort

### Follow-up

Participant data was extracted for the 12-year period from 2006 to 2017, inclusive. There were 1,107,481 eligible patients in the first year of the cohort (2006), increasing to 1,491,954 at the beginning of 2017, with a total of 1,070,175 new participants joining the cohort between 2007 and 2017. Patients contributed a mean of 5 years of data, with 647,239 patients (58.4%) who were present in the first cohort year (2006) having the full 12 years of data. Patients present in 2006 comprised 50.9% of the cohort and contributed 67.0% of the total person-years.

**Fig. 2:**
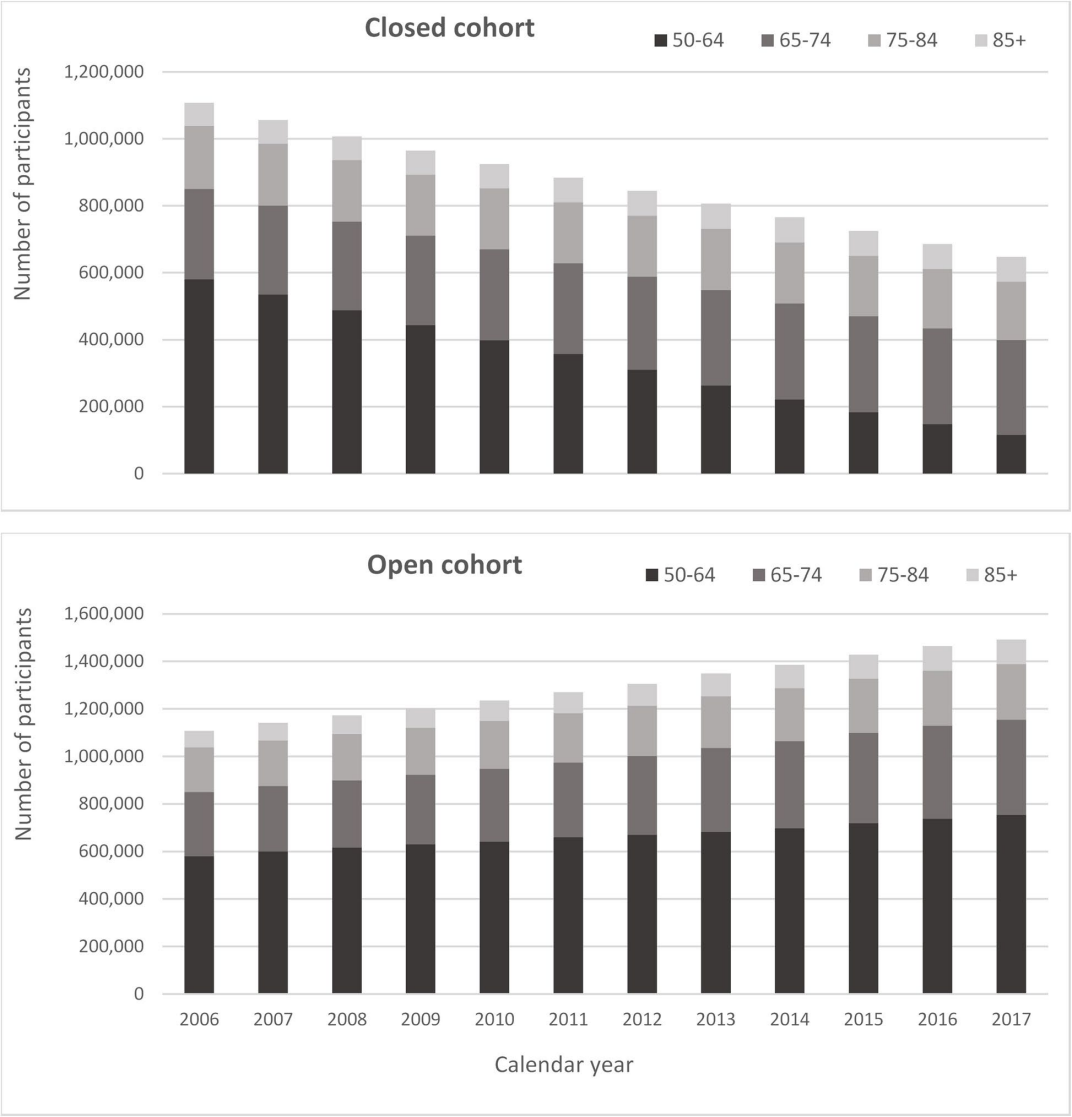
Age group distribution over cohort period

Between 2006 and 2017, 137,481 patients died (6.3% of cohort) and 635,400 patients moved out of an RCGP RSC practice (29.2% of the cohort). The full details of entry and exit to the cohort by calendar year according to age groups and frailty category at cohort entry are given in Supplemental Tables 1 and 2. There was an inflow of new participants over the cohort period, across all age groups and frailty categories, which was more notable in younger age groups. The mean follow-up period increased from 4.8 years in people categorised as fit at cohort entry to 7.4 years in people categorised as severely frail (Supplemental Table 3). The age distribution over the cohort period for the closed cohort (participants who were present in 2006 onwards, showing attrition due to death and leaving RCGP RSC practices) and the open cohort (participants present in 2006 plus those moving into an RCGP RSC practice and people turning 50) is given in Fig. 2.

## Discussion

There is an urgent need for a better understanding of current and future care requirements for people with frailty, including those in middle age to the younger old, where there is likely to be unmet need. This cohort of approximately 2.1 million adults aged 50 and over with long-term follow-up data on frailty status using the electronic Frailty Index (eFI) applied to routinely collected primary care data is the largest cohort so far that will be used for longitudinal analysis to explore frailty dynamics and its impact using linked hospital and mortality data. The dataset includes adults aged 50 and over from around 5% of general practices from a single country (England), thus meeting the project aims to provide a whole-system analysis of representative population-level data. The general practices from which the cohort is derived vary in their characteristics, with a range of geographical locations, urban/rural mix, practice sizes and areas of differing deprivation, further demonstrating the representativeness and generalisability of the RCGP RSC dataset [19]. This diversity will reflect a variety of care settings and approaches to managing people with frailty, so that the subsequent simulation models can reflect population and care heterogeneity.

Characteristics of patients at cohort entry show expected patterns in sociodemographic variables and trends in clinical conditions and lifestyle factors, further demonstrating representativeness of the data and suitability for planned analyses. The observed trends in increasing eFI with age group and the greater proportions of moderate and severe frailty categories in older age groups reflect current knowledge, and the observed presence of frailty in 10% of adults aged 50–64 in our cohort highlights the importance of examining frailty and its trajectories earlier in life. The study design of an open cohort allows entry of younger patients throughout its duration, representing a dynamically ageing population in which the overall mean age increases over the cohort period. The cohort therefore includes substantial data on middle-aged adults which is novel, and also crucial for observing potential earlier manifestations of frailty and its progression over time, as it is likely that future interventions to reduce incidence and progression to earlier stages of frailty may be targeted at this age group.

### Study strengths and limitations

This dataset is derived from a single national health service (NHS), in which registered patients are managed in accordance with specific guidelines and broadly similar care pathways even though living in different regions of the country and under different primary healthcare practices. Primary care is free at the point of delivery, as is hospital care. GP registration coverage in England is high [32] therefore data from the primary care population are essentially representative of the overall population. However, the cohort does not include information from private healthcare, which is most commonly available via private medical insurance and accessed by around 11% of the UK population, although schemes have limited cover for general practice [33]. Information on adult social care, which is means tested, is also not available as it is organised via a mix of state, private and voluntary providers.

The large size of the dataset enables the stratification of the cohort by key characteristics whilst maintaining sufficiently sized subgroups to provide precise estimates to inform services for geographical areas with particular population characteristics. The dataset includes a wide range of covariates which have been identified in other studies as being associated with either frailty onset or outcomes following frailty occurrence. However, covariates such as social factors (e.g. loneliness, living situation) and contemporaneous information on residential care status is not available. To address this, information on the impact of such factors on outcomes will be sourced from the literature and included within ongoing analysis supporting the development of the simulation model. Large EHR datasets will necessarily reflect local differences in data entry and coding procedures, and accuracy of the eFI will also depend on timely attendance of patients to healthcare services to enable assessment and diagnosis, and on transfer of information between different healthcare services. The cumulative nature of the eFI and its validation in large routine healthcare datasets from different countries [34–36] demonstrate that the eFI is capable of identifying frailty as a vulnerability, and suggests variability in coding accuracy or completeness does not have a significant impact on the ability of the eFI to identify changes in frailty status in ageing populations.

Approximately one third of participants have no ethnicity data, which is self-reported administrative data more prone to be missing than clinical data. There appears to be under-reporting of non-white ethnicity, with participants identifying as ‘white’ comprise 92% of the given values, as compared to 86% in the 2011 Census (data for England and Wales) [37]. It is possible that the under-representation of people from ethnic minorities could also be due to recognised issues with lower primary healthcare usage, rather than practices not reflecting their catchment populations, or due to a reporting bias in the primary care data [38]. However, inclusion of ethnicity data from HES in future planned analyses should increase the proportion of the cohort with recorded ethnicity [39].

During the cohort period, there was significant movement of participants both into and out of the cohort, reflecting real-life population flows which are key parameters for simulation modelling of population health needs. Frailty data and patient and service use outcomes are being collected for each year that the patient is registered, thus providing full outcome ascertainment for each year of participation. The 65–74 group had the lowest number of exits, and higher numbers in other age groups could be a consequence of greater mobility in the working age population or moves related to higher levels of support in older age groups, for example following a health or social care crisis [40]. This could lead to an underestimation of incidence and progression of frailty in our cohort, although participants of older age and greater frailty severity also had the longest follow-up periods. Although the location of practices seemed equally distributed across the deprivation quintiles, there was a trend across all age groups for greater representation of patients from the least deprived quintiles, perhaps reflecting movement to wealthier retirement areas and the suburbs.

The 36 eFI deficits and other long-term conditions were defined using Read codes. For future data extractions, migration of clinical term definitions from Read codes to the Systemised Nomenclature of Medicine – Clinical Terms (SNOMED CT®) will be necessary to reflect national harmonisation of coding tools across the healthcare pathway [41].

### Summary of planned analyses

This dataset will be linked with outcome data on secondary care service use from NHS Digital so that population-level parameters for the simulation model, for example the numbers of hospital admissions for people in different frailty states in each calendar year, can be calculated. The long follow-up period, averaging 5 years and with more than 600,000 participants over 12 years, is essential for being able to move beyond exploring impact of frailty states and associated outcomes at a defined timepoint. The time frame allows for many transitions between frailty states over the cohort period to be observed and gives ample scope for investigating frailty trajectories in the population to inform population-level planning for required services.

Epidemiological and statistical techniques will be employed to describe the incidence and prevalence of frailty over the cohort period and within population subgroups and rates of transitions between frailty states. Relationships between patient characteristics and frailty transitions and associations between frailty states and outcomes, including hospitalisations, mortality and service use, will be explored. Costs will be attributed to service use and predictions of future service use and costs under different scenarios will be produced using the simulation model.

Current evidence suggests that socioeconomic and educational deprivation is associated with higher frailty scores [42]. Analysis of this cohort will enable an in-depth analysis of the patterns of frailty onset and transitions according to social deprivation, which will inform decisions on whether this potentially high-risk population may benefit from targeted earlier intervention to improve outcomes.

The geographical range of the data will enable parameterisation of the simulation model with more localised data, permitting adaptation to reflect local situations and facilitating planning on whichever scale is the most appropriate. If there is interest in looking at the data for particular GP characteristics, for example focussing on larger practices or practices positioned in more deprived areas, the primary analyses can be re-run for the required subgroups to adjust parameters for the simulation model.

## Conclusions

This cohort is the largest dataset compiling frailty transitions and outcomes and will provide unique information on frailty within middle-aged to young-old populations. Data presented here show that frailty is already common in middle-age and continues to increase across the later life course. The cohort will allow exploration of the impact of morbidities, socioeconomic and lifestyle factors on frailty onset, trajectories and outcomes over time. Strengths of this cohort are the use of large-scale routine primary care data with linkage to secondary care and healthcare costs, and a dynamically ageing population with lengthy follow-up, enabling novel insights into the onset and progression of frailty.

## Supplementary Information

**Additional file 1: Supplemental Table 1.** Entry and exit to the cohort in each calendar year by age group. **Supplemental Table 2.** Entry and exit to the cohort in each calendar year by frailty category. **Supplemental Table 3.** Mean length of follow-up by age group and frailty category.

## Data Availability

The data that support the findings of this study are available from the RCGP RSC and NHS Digital, but restrictions apply to the availability of these data, which were used following approvals and data sharing agreements for the current study, and so are not publicly available.

## Abbreviations

BMI: Body mass index
BNF: British National Formulary
CTV3: Clinical Terms Version 3
DARS: Digital Access Request Service
eFI: Electronic Frailty Index
EHR: Electronic Health Records
FTE: Full-time equivalent
GP: General Practitioner
HES: Hospital Episode Statistics
HSDR: Health Services & Delivery Research
IDAOPI: Income Deprivation Affecting Older People Index
IGARD: Independent Group Advising on the Release of Data
IMD: Indices of Multiple Deprivation
NHS: National Health Service
NIHR: National Institute of Health Research
ONS: Office for National Statistics
PYAR: Person-years at risk
QOF: Quality Outcomes Framework
RCGP RSC: Royal College of General Practitioners Research Surveillance Centre
RUC11: Rural urban classification 11
SD: Standard deviation
SEG: Stakeholder Engagement Group
SNOMED CT®: Systemised Nomenclature of Medicine – Clinical Terms

## References

[1] Clegg A, Young J, Iliffe S, Rikkert MO, Rockwood K (2013). Frailty in elderly people. Lancet.

[2] Campbell AJ, Buchner DM (1997). Unstable disability and the fluctuations of frailty. Age Ageing.

[3] Fried LP, Tangen CM, Walston J, Newman AB, Hirsch C, Gottdiener J, Seeman T, Tracy R, Kop WJ, Burke G (2001). Frailty in older adults: evidence for a phenotype. J Gerontol A Biol Sci Med Sci.

[4] Aguayo GA, Donneau AF, Vaillant MT, Schritz A, Franco OH, Stranges S, Malisoux L, Guillaume M, Witte DR (2017). Agreement between 35 published frailty scores in the general population. Am J Epidemiol.

[5] O'Caoimh R, Galluzzo L, Rodriguez-Laso A, Van der Heyden J, Ranhoff AH, Lamprini-Koula M, Ciutan M, Lopez-Samaniego L, Carcaillon-Bentata L, Kennelly S (2018). Prevalence of frailty at population level in European ADVANTAGE joint action member states: a systematic review and meta-analysis. Ann Ist Super Sanita.

[6] Buchman AS, Wilson RS, Bienias JL, Bennett DA (2009). Change in frailty and risk of death in older persons. Exp Aging Res.

[7] Stolz E, Mayerl H, Freidl W (2019). Fluctuations in frailty among older adults. Age Ageing.

[8] Welstead M, Jenkins ND, Russ TC, Luciano M, Muniz-Terrera G. A systematic review of frailty trajectories: their shape and influencing factors. The Gerontologist. 2021;61(8):e463–75. 10.1093/geront/gnaa061 PMC8599181 32485739

[9] Hoogendijk EO, Rockwood K, Theou O, Armstrong JJ, Onwuteaka-Philipsen BD, Deeg DJH, Huisman M (2018). Tracking changes in frailty throughout later life: results from a 17-year longitudinal study in the Netherlands. Age Ageing.

[10] Stolz E, Mayerl H, Hoogendijk EO, Armstrong JJ, Roller-Wirnsberger R, Freidl W (2021). Acceleration of health deficit accumulation in late-life: evidence of terminal decline in frailty index three years before death in the US health and retirement study. Ann Epidemiol.

[11] Bai G, Szwajda A, Wang Y, Li X, Bower H, Karlsson IK, et al. Frailty trajectories in three longitudinal studies of aging: is the level or the rate of change more predictive of mortality? Age Ageing. 2021;50(6):2174–82. 10.1093/ageing/afab106 PMC8581383 34120182

[12] Rohrmann S, Veronese N (2020). Epidemiology of Frailty in Older People. Frailty and Cardiovascular Diseases, Advances in Experimental Medicine and Biology, 1216.

[13] Hollinghurst J, Fry R, Akbari A, Clegg A, Lyons RA, Watkins A, et al. External validation of the electronic frailty index using the population of Wales within the secure Anonymised information linkage databank. Age Ageing. 2019. 10.1093/ageing/afz110 PMC6814149 31566668

[14] Clegg A, Bates C, Young J, Ryan R, Nichols L, Ann Teale E, Mohammed MA, Parry J, Marshall T (2016). Development and validation of an electronic frailty index using routine primary care electronic health record data. Age Ageing.

[15] NHS England (2018). NHS standard general medical services contract 2017/18.

[16] NHS England (2019). The NHS long term plan.

[17] Walsh B (2018). Study protocol: the dynamics of frailty in older people: modelling impact on health care demand and outcomes to inform service planning and commissioning.

[18] Brailsford SC, Lattimer VA, Tarnaras P, Turnbull JC (2004). Emergency and on-demand health care: modelling a large complex system. J Oper Res Soc.

[19] Correa A, Hinton W, McGovern A, van Vlymen J, Yonova I, Jones S, de Lusignan S (2016). Royal College of general practitioners research and surveillance Centre (RCGP RSC) sentinel network: a cohort profile. BMJ Open.

[20] Hinton W, McGovern A, Coyle R, Han TS, Sharma P, Correa A, Ferreira F, de Lusignan S (2018). Incidence and prevalence of cardiovascular disease in English primary care: a cross-sectional and follow-up study of the Royal College of general practitioners (RCGP) research and surveillance Centre (RSC). BMJ Open.

[21] HM Government (2015). File 3: supplementary indices - income deprivation affecting children index and income deprivation affected older people index.

[22] Office for National Statistics (2017). Output Area to LSOA to MSOA to Local Authority District (December 2017) Lookup with Area Classifications in Great Britain.

[23] NHS Digital: General practice workforce. https://digital.nhs.uk/data-and-information/publications/statistical/general-and-personal-medical-services.

[24] Searle SD, Mitnitski A, Gahbauer EA, Gill TM, Rockwood K (2008). A standard procedure for creating a frailty index. BMC Geriatr.

[25] Department for Communities and Local Government. The English Index of Multiple Deprivation (IMD) 2015 – Guidance: Department for Communities and Local Government; 2015. https://www.gov.uk/government/statistics/english-indices-of-deprivation-2015.

[26] Tippu Z, Correa A, Liyanage H, Van Vlymen J, Burleigh D, McGovern A, Jones S, de Lusignan S (2016). Ethnicity recording in primary care computerised medical record systems: an ontological approach. BMJ Health Care Inform.

[27] Office for National Statistics (2011). 2011 rural/urban classification (RUC11).

[28] NHS. Digital: general and personal medical services, practice level dataset: Digital N; 2013. https://digital.nhs.uk/data-and-information/publications/statistical/general-and-personal-medical-services/2003-2013-as-at-30-september.

[29] Joy M, Hobbs FDR, McGagh D, Akinyemi O, de Lusignan S. Excess mortality from COVID-19 in an English sentinel network population. Lancet Infect Dis. 2020;21(4):e74. 10.1016/S1473-3099(20)30632-0 PMC7402647 32763192

[30] Maguire A, Blak BT, Thompson M (2009). The importance of defining periods of complete mortality reporting for research using automated data from primary care. Pharmacoepidemiol Drug Saf.

[31] Joint Formulary Committee (2020). British National Formulary.

[32] Burch P, Doran T, Kontopantelis E (2018). Regional variation and predictors of over-registration in English primary care in 2014: a spatial analysis. J Epidemiol Community Health.

[33] The King's Fund (2014). The UK private health market.

[34] Millares-Martin P (2019). Large retrospective analysis on frailty assessment in primary care: electronic frailty index versus frailty coding. BMJ Health Care Inform.

[35] Devereux N, Ellis G, Dobie L, Baughan P, Monaghan T (2019). Testing a proactive approach to frailty identification: the electronic frailty index. BMJ Open Qual.

[36] Boyd PJ, Nevard M, Ford JA, Khondoker M, Cross JL, Fox C (2018). The electronic frailty index as an indicator of community healthcare service utilisation in the older population. Age Ageing.

[37] Ethnicity facts and figures [https://www.ethnicity-facts-figures.service.gov.uk/uk-population-by-ethnicity/national-and-regional-populations/population-of-england-and-wales/latest ].

[38] Lakhani M. No patient left behind: how can we ensure world class primary care for black and minority ethnic people? London: Department of Health; 2008.

[39] Mathur R, Bhaskaran K, Chaturvedi N, Leon DA, vanStaa T, Grundy E, Smeeth L (2014). Completeness and usability of ethnicity data in UK-based primary care and hospital databases. J Public Health (Oxf).

[40] Scheibl F, Farquhar M, Buck J, Barclay S, Brayne C, Fleming J, Collaboration obotCCo-sCS (2019). When frail older people relocate in very old age, who makes the decision?. Innov Aging.

[41] HM Government. Personalised health and care 2020. In: Using Data and Technology to Transform Outcomes for Patients and Citizens. A Framework for Action. London: Board NI; 2014.

[42] Franse CB, van Grieken A, Qin L, Melis RJF, Rietjens JAC, Raat H (2017). Socioeconomic inequalities in frailty and frailty components among community-dwelling older citizens. PLoS One.

