# Supplementary material for: The dynamics of frailty development and progression in older adults in primary care in England (2006–2017): a retrospective cohort profile
Source: BMC Geriatr. 2022 Jan 6;22:30. doi: 10.1186/s12877-021-02684-y (PMC8740419; doi:10.1186/s12877-021-02684-y)
Supplement: Supplementary file 1 — Additional file 1: Supplemental Table 1. Entry and exit to the cohort in each calendar year by age group. Supplemental Table 2. Entry and exit to the cohort in each calendar year by frailty category. Supplemental Table 3. Mean length of follow-up by age group and frailty category. [file 12877_2021_2684_MOESM1_ESM.docx]

**Supplemental Table 1. Entry and Exit to the cohort in each calendar year by age group**

| **Calendar year** | **Age group*** | | | | | | | |
| --- | --- | --- | --- | --- | --- | --- | --- | --- |
|  | **50-64** | | **65-74** | | **75-84** | | **85+** | |
|  | **Entry** | **Exit** | **Entry** | **Exit** | **Entry** | **Exit** | **Entry** | **Exit** |
| 2006** | 579,593 | 17,176 | 270,326 | 9,538 | 187,870 | 12,951 | 69,692 | 12,071 |
| 2007 | 65,363 | 18,432 | 9,550 | 9,741 | 6,226 | 12,755 | 3,875 | 12,484 |
| 2008 | 66,678 | 17,474 | 8,749 | 9,158 | 5,977 | 12,567 | 3,864 | 13,255 |
| 2009 | 65,529 | 16,868 | 7,629 | 9,224 | 5,393 | 12,495 | 3,951 | 13,766 |
| 2010 | 66,949 | 18,866 | 8,237 | 10,285 | 5,410 | 12,644 | 4,003 | 13,875 |
| 2011 | 71,990 | 20,515 | 8,792 | 10,644 | 5,924 | 12,619 | 4,192 | 14,326 |
| 2012 | 74,235 | 21,278 | 9,413 | 11,248 | 5,958 | 13,211 | 4,330 | 15,562 |
| 2013 | 81,143 | 24,506 | 11,940 | 12,974 | 6,877 | 14,765 | 4,788 | 17,518 |
| 2014 | 81,922 | 27,413 | 11,598 | 14,034 | 6,887 | 15,200 | 5,039 | 17,475 |
| 2015 | 88,893 | 27,446 | 14,398 | 14,589 | 8,322 | 15,637 | 5,586 | 18,953 |
| 2016 | 86,650 | 29,270 | 12,638 | 15,587 | 7,499 | 15,966 | 5,425 | 19,344 |
| 2017 | 84,631 | 31,297 | 12,204 | 17,196 | 6,782 | 16,856 | 4,736 | 20,477 |
| **Total** | **1,413,576** | **270,541** | **385,474** | **144,218** | **259,125** | **167,666** | **119,481** | **189,106** |

*Note: people move between age group categories during cohort period

**Entry cells in 2006 denote people registered in an RCGP practice at the beginning of the cohort period

**Supplemental Table 2. Entry and Exit to the cohort in each calendar year by frailty category**

| **Calendar year** | **Frailty Category*** | | | | | | | |
| --- | --- | --- | --- | --- | --- | --- | --- | --- |
|  | **Fit** | | **Mild** | | **Moderate** | | **Severe** | |
|  | **Entry** | **Exit** | **Entry** | **Exit** | **Entry** | **Exit** | **Entry** | **Exit** |
| 2006** | 812,788 | 28,259 | 226,987 | 14,630 | 55,885 | 6,604 | 11,821 | 2,243 |
| 2007 | 73,340 | 27,796 | 9,842 | 14,898 | 1,639 | 7,701 | 193 | 3,017 |
| 2008 | 73,357 | 25,289 | 9,985 | 14,738 | 1,707 | 8,641 | 219 | 3,786 |
| 2009 | 70,460 | 24,460 | 9,875 | 14,604 | 1,906 | 9,068 | 261 | 4,221 |
| 2010 | 71,270 | 25,971 | 10,892 | 15,183 | 2,152 | 9,647 | 285 | 4,869 |
| 2011 | 76,277 | 27,151 | 11,880 | 15,404 | 2,388 | 10,319 | 353 | 5,230 |
| 2012 | 78,432 | 27,492 | 12,471 | 16,298 | 2,580 | 11,268 | 453 | 6,241 |
| 2013 | 86,412 | 31,918 | 14,585 | 18,049 | 3,164 | 12,533 | 587 | 7,263 |
| 2014 | 87,092 | 34,722 | 14,701 | 18,614 | 3,104 | 12,873 | 549 | 7,913 |
| 2015 | 95,372 | 33,935 | 17,053 | 19,488 | 3,961 | 14,011 | 813 | 9,191 |
| 2016 | 91,287 | 36,417 | 16,207 | 19,645 | 3,823 | 14,226 | 895 | 9,879 |
| 2017 | 87,511 | 37,542 | 15,455 | 21,268 | 4,005 | 15,374 | 1,382 | 11,642 |
| **Total** | **1,703,598** | **360,952** | **369,933** | **202,819** | **86,314** | **132,265** | **17,811** | **75,495** |

*Note: people move between frailty categories during cohort period

**Entry cells in 2006 denote people registered in an RCGP practice at the beginning of the cohort period

**Supplemental Table 3. Mean length of follow-up by age group and frailty category**

|  | **Number of participants** | **Mean years of follow-up** |
| --- | --- | --- |
| **Age group** |  |  |
| 50-64 | 1,413,576 | 4.4 |
| 65-74 | 385,474 | 6.2 |
| 75-84 | 259,125 | 6.1 |
| 85+ | 119,481 | 6 |
| **Frailty category** |  |  |
| Fit | 1,703,598 | 4.8 |
| Mild | 369,933 | 5.9 |
| Moderate | 86,214 | 6.6 |
| Severe | 17,811 | 7.4 |
